# Dietary Supplementation with *Eucommia ulmoides* Leaf Extract Improved the Intestinal Antioxidant Capacity, Immune Response, and Disease Resistance against *Streptococcus agalactiae* in Genetically Improved Farmed Tilapia (GIFT; *Oreochromis niloticus*)

**DOI:** 10.3390/antiox11091800

**Published:** 2022-09-13

**Authors:** Dongyu Huang, Jian Zhu, Lu Zhang, Xianping Ge, Mingchun Ren, Hualiang Liang

**Affiliations:** 1 Wuxi Fisheries College, Nanjing Agricultural University, Wuxi 214081, China; 2 Key Laboratory of Integrated Rice-Fish Farming Ecology, Ministry of Agriculture and Rural Affairs, Freshwater Fisheries Research Center, Chinese Academy of Fishery Sciences, Wuxi 214081, China; 3 Tongwei Co., Ltd., Healthy Aquaculture Key Laboratory of Sichuan Province, Chengdu 610093, China

**Keywords:** *Eucommia ulmoides* leaf extract, antioxidant capacity, immune response, apoptosis, disease resistance, GIFT

## Abstract

A 7-week rearing trial was designed to investigate the effects of *Eucommia ulmoides* leaf extract (ELE) on growth performance, body composition, antioxidant capacity, immune response, and disease susceptibility of diet-fed GIFT. The results showed that dietary ELE did not affect growth performance or whole-body composition (*p* > 0.05). Compared with the control group, plasma ALB contents increased in the 0.06% dietary ELE group (*p* < 0.05), and plasma ALT and AST activities decreased in the 0.08% dietary ELE group (*p* < 0.05). In terms of antioxidants, compared with GIFT fed the control diet, 0.06% dietary ELE upregulated the mRNA expression levels of Nrf2 pathway-related antioxidant genes, including *CAT* and *SOD* (*p* < 0.05), and 0.06% and 0.08% dietary ELE upregulated the mRNA levels of *Hsp70* (*p* < 0.05). In terms of immunity, 0.06% dietary ELE suppressed intestinal *TLR2*, *MyD88*, and *NF-κB* mRNA levels (*p* < 0.05). Moreover, the mRNA levels of the anti-inflammatory cytokines *TGF-β* and *IL-10* were upregulated by supplementation with 0.04% and 0.06% dietary ELE (*p* < 0.05). In terms of apoptosis, 0.06% and 0.08% ELE significantly downregulated the expression levels of *FADD* mRNA (*p* < 0.05). Finally, the challenge experiment with *S. agalactiae* showed that 0.06% dietary ELE could inhibit bacterial infection, and significantly improve the survival rate of GIFT (*p* < 0.05). This study demonstrated that the supplementation of 0.04–0.06% ELE in diet could promote intestinal antioxidant capacity, enhance the immune response and ultimately improve the disease resistance of GIFT against *Streptococcus agalactiae*.

## 1. Introduction

Tilapia (*Oreochromis niloticus*) is the most exported farmed fish in China, with a total production of 1.65 million tons farmed in 2020 [1]. Nevertheless, in recent years, outbreaks of streptococcal disease have caused significant losses in the tilapia industry. Currently, *Streptococcus* is an important pathogenic bacterium that affects tilapia, and the disease is likely to occur when the water temperature is above 31 °C [2]. Usually, tilapia is more resistant to diseases; however, the seasonal high temperatures in summer are probably the cause of low immunity in tilapia and the increase in the susceptibility of these fish to pathogenic bacteria, resulting in a significant increase in mortality due to streptococcal infection [3,4]. Thus, to improve the ability of tilapia to fight bacterial infection, additives (such as *Bacillus pumilus* and white button mushrooms) have been applied to enhance immunocompetence and antioxidative status [5,6]. The use of feed additives to improve the immunity and disease resistance of tilapia has become a major trend worthy of further research to provide a reference for the tilapia industry.

*Eucommia ulmoides* is an endemic plant species in China. The leaves and bark of *E. ulmoides* can be used as a growth promoter for animals, with growth-supporting, lactation, and immune-enhancing effects [7]. As a byproduct of the traditional Chinese herb *E. ulmoides*, *E. ulmoides* leaves are quite common in China [8] and show higher antioxidative activity than the cortex, fruits, and flowers [9]. In recent years, experts have studied the composition and efficacy of *E. ulmoides* leaves, they concluded that *Eucommia ulmoides* leaf extract (ELE) as a feed additive showed no drug resistance and almost no toxic side effects [10,11], indicating that ELE is a very valuable feed additive for development. Furthermore, ELE is rich in bioactive compounds (e.g., flavonoids, chlorogenic acid, peach leaf coralline, kynurenine) with anti-inflammatory, antioxidant, antiviral, and hepatoprotective properties [12]. In recent years, several researchers have reported that ELE increases the body weight of weaned piglets [13] and broiler chickens [14] and increases the feed intake of piglets [15]. Moreover, in a study on lambs, it was found that the addition of extracts from *Eucommia ulmoides* leaves in the diet did not affect their average daily weight gain or feed efficiency [16], which may be related to the amount of ELE added and the particular animal species. In aquatic animals, Huang et al. [17] found that 1.0% dietary ELE supplementation could improve the growth performance of large yellow croaker (*Larimichthys crocea*) and enhance antioxidant capacity and immunity. Zhang et al. [18] studied channel catfish (*Ictalurus punctatus*) and reported that 0.2% dietary ELE supplementation improved the intestinal microbiota structure and reduced the incidence of disease. The above studies show that ELE is increasingly being used in aquaculture, but its use as an additive for fish is still relatively rare, and the immunomodulatory regulatory mechanisms of ELE deserve further study.

Toll-like receptor (TLR) family-mediated innate immunity is the first line of defense against disease [19]. TLRs are the primary receptors for the recognition of pathogen-associated molecular patterns (PAMPs) by the innate immune system that initiate the signaling pathways that regulate the adaptive immune response [20]. In addition, the TLRs can bind myeloid differentiation factor 8 (*MyD88*) to activate nuclear factor kappa-B (*NF-κB*) and apoptotic signaling pathways [21,22]. In aquatic animals, many studies have indicated that *TLR2* plays a critical role in the innate immune response [23,24]. However, no information regarding the effects of ELE on the immune response and apoptosis related to the *TLR2*-*MyD88* pathway in tilapia has been reported. Additionally, the nuclear factor erythroid 2-related factor 2 (*Nrf2*) signaling pathway plays a critical role in the resistance to exogenous or endogenous oxidative stress [25]. Likewise, the mechanism by which ELE regulates the antioxidant status of tilapia via the *Nrf2* signaling pathway deserves investigation.

In this study, the genetically improved farmed tilapia (GIFT), one of the tilapia strains, was chosen as the subject of this experiment. GIFT was developed in response to the growing demand for superior growth rates and increased resistance to emerging diseases among fish in aquaculture through international efforts [26]. The GIFT is now reportedly being cultured in about 87 countries around the world [1] and is one of the most popular aquaculture species in China. Thus, the objectives of our study were to examine the effects of ELE on growth, antioxidant capacity associated with the *Nrf2* signaling pathway, immune response and apoptosis induction associated with the *TLR2*-*MyD88* signaling pathway, and the disease resistance of GIFT against *Streptococcus agalactiae*.

## 2. Materials and methods

### 2.1. Diet Preparation

Table 1 shows the ingredients and proximate composition of the experimental diets. ELE was purchased from HANOVE Animal Health Products Co., Ltd., Wuxi, China. According to the recommended dosage (0.03–0.06%) of this product in omnivorous fish feed, the ELE was supplemented in the diet at five levels (0% (control), 0.02%, 0.04%, 0.06%, and 0.08%). All of the ingredients used in this experiment were crushed and passed through a 60-mesh sieve, made into pellets (the grain diameter is 1.0 mm), and then dried in an oven at 45 °C for 24 h. The specific steps and instruments used were described in our previous report [27]. After drying, the pellets were put into self-sealing bags and stored at −20 °C until further use.

### 2.2. Experimental Fish and Procedures

GIFT juveniles were provided by the breeding farm of the Freshwater Fisheries Research Center (FFRC) of the Chinese Academy of Fishery Sciences (Wuxi, China). Before the experiment, all fish were temporarily reared in floating cages for two weeks to adapt to the experimental environment. Afterward, 300 healthy fish (initial body weight was 12.04 ± 0.03 g) were randomly assigned to 15 floating cages (1 m × 1 m × 1 m) (20 fish per cage). Each diet consisted of three replicates. The experiment lasted for 7 weeks, during which the fish were fed twice a day, each time to apparent satiety. Additionally, the water quality indicators were recorded daily (YSI ProDSS Multiparameter Water Quality Meter, Ohio, USA), the water temperature was maintained between 31 and 33 °C, the amount of dissolved oxygen was higher than 6 mg/L, and the pH was kept at 7.0–7.5.

### 2.3. Sample Collection

After 7 weeks, the experimental fish were fasted for 24 h, after which the number of GIFT per cage was counted, and all fish were weighed. Three fish were randomly taken from each cage. First, blood was drawn from the caudal vein and immediately centrifuged for 10 min (3000 rpm, 4 °C). Then, upper plasma samples were obtained and stored in a -20 °C freezer for plasma biochemical analysis. Intestinal samples were collected by dissection. A portion of the intestinal tissue was stored in 4% paraformaldehyde for pathological analysis, and the remaining intestinal samples were stored in a −80 °C freezer for gene and enzymatic activity analysis.

### 2.4. Proximate Composition and Chemical Analysis

The experimental diets and whole-body composition were analyzed based on the method of AOAC [28]. Plasma total protein (TP), albumin (ALB), alanine aminotransferase (ALT), and aspartate aminotransferase (AST) were determined with an automatic biochemical analyzer. The intestinal activities of antioxidant factors (malondialdehyde (MDA), superoxide dismutase (SOD), catalase (CAT), glutathione (GSH), and glutathione peroxidase (GPx)) were analyzed with the corresponding reagent kits. The major kits, testing equipment, and main methods are presented in Table 2.

### 2.5. Histology

Hematoxylin and eosin (HE) staining was used to analyze the intestinal histology. First, the intestinal tissue samples were extracted from 4% paraformaldehyde. Then, intact wax blocks were obtained by gradient alcohol dehydration and embedding, followed by serial sectioning with a microtome (Leica Company, Wetzlar, Germany), HE staining and dehydration sealing. Finally, a Zeiss microscope (Axioplan-2, Oberkochen, Germany) was used to observe the intestinal pathological changes, and photographs were collected for analysis

### 2.6. Real-Time PCR Analysis

First, the TRIzol method (Vazyme Biotech Co., Ltd., Nanjing, China) was used to extract total RNA from the intestinal tissues. Then, the quality and quantity of the RNA were checked with a NanoDrop 2000 spectrophotometer. Finally, the reaction system was set up according to the instructions of the HiScript^®^ II One Step qRT-PCR SYBR Green Kit (Q221-01, Vazyme, Nanjing, China) and performed on a CFX96 real-time PCR detection system thermocycler (Bio-Rad). The specific primers for the reference gene (*β-actin*) and target genes in this experiment are displayed in Table 3. The mRNA expression levels were calculated from the standard curve, normalized to *β-actin*, and quantified using the relative standard curve method.

### 2.7. Streptococcus Agalactiae Challenge Test

Ten fish from each cage were challenged with *Streptococcus agalactiae* (*S. agalactiae*) in indoor recirculating culture barrels with a controlled water temperature at 32 ± 1 °C, the pH value ranged from 7.6 ± 0.2, and dissolved oxygen levels were maintained at 6–7 mg/L. Before the challenge, a pre-experiment was performed to determine the half-lethal concentration (1 × 10^6^ CFU/mL) of *S. agalactiae* using a bacterial turbidimeter (SGZ-6AXJ, Yue Feng Instrument Co., Ltd., Shanghai, China). The specific method is described in our previous study [29]. Then, the fish were challenged by intraperitoneal injection with 1 mL/100 g (1% of body weight). The mortality rate within 144 h was recorded.

### 2.8. Statistical Analysis

The data were subjected to normality and homogeneity tests. Then, the experimental data (means ± SEMs) were analyzed using SPSS 24.0 statistical software for one-way analysis of variance (ANOVA). When the difference was significant (*p* < 0.05), Duncan’s multiple comparisons tests were performed. Furthermore, orthogonal polynomial contrasts were used to assess the significance of linear or quadratic models to describe the response of the dependent variable to dietary ELE levels. *p*-values < 0.05 were considered statistically significant.

## 3. Results

### 3.1. Growth Performance and Whole-Body Composition

Table 4 shows the GIFT growth performance results. The FBW, FCR, WGR, SGR, and SR were not influenced by dietary ELE levels (*p* > 0.05). Table 5 presents the whole-body composition of the GIFT, and no significant effect of ELE supplementation was found on the moisture, protein, lipid, and ash content in all diets (*p* > 0.05).

### 3.2. Plasma Parameters

The results of the plasma parameter assessment of the GIFT fed different diets are presented in Table 6. Plasma ALB had a positive linear with increasing dietary ELE inclusion levels (*p* < 0.05). At a dietary ELE level of 0.06%, plasma ALB showed the highest level (*p* < 0.05). In addition, both plasma ALT and AST had negative linear responses with increasing dietary ELE inclusion levels (*p* < 0.05), and plasma ALT activity of the fish fed 0.08% dietary ELE were lower than those fed the control diet (*p* < 0.05). The plasma AST activities of the fish fed 0.06% and 0.08% dietary ELE were lower than those fed the control diet (*p* < 0.05). Plasma TP levels were not influenced by dietary ELE levels (*p* > 0.05).

### 3.3. Intestinal Antioxidant Enzyme Activities

Table 7 shows the results of intestinal antioxidant enzyme activities of the GIFT fed different diets. The CAT and SOD had an open upward parabola with increasing dietary ELE inclusion levels (*p* < 0.05). The highest CAT and SOD activity was observed in the 0.04% and 0.06% ELE groups, respectively, which were notably higher than those in the group administered the control diet (*p* < 0.05). The GSH-Px had a positive linear response with increasing dietary ELE inclusion levels (*p* < 0.05), and at a dietary ELE level of 0.06%, GSH-Px showed the highest activity (*p* < 0.05). In addition, the GSH had an open upward parabola with increasing dietary ELE inclusion levels (*p* < 0.05), but no significant differences in GSH content were observed among all groups (*p* > 0.05). Furthermore, dietary ELE levels did not affect GSH and MDA contents (*p* > 0.05).

### 3.4. Histopathological Examination

**Figure 1:**
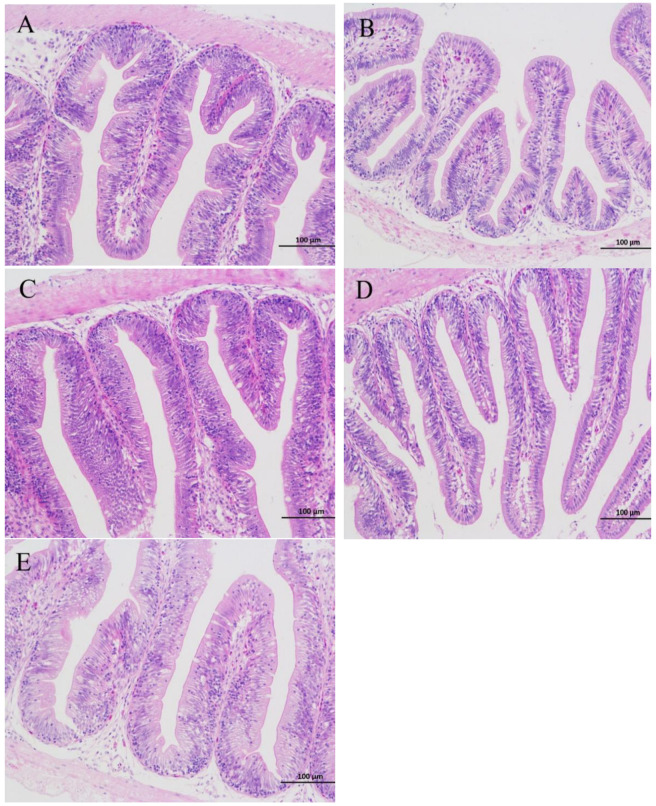
The intestine sections HE staining of the GIFT with different ELE levels (200×). 0% ELE (**A**), 0.02%ELE (**B**), 0.04%ELE (**C**), 0.06%ELE (**D**), and 0.08%ELE (**E**).

Figure 1 shows photomicrographs of intestinal sections, and the data on the number of goblet cells and villus length are shown in Table 8. When the ELE inclusion level was 0.06%, the number of goblet cells was significantly larger than that in the control group (*p* < 0.05). In addition, no significant effect of ELE supplementation was found on villus length compared with the control group (*p* > 0.05).

### 3.5. Nrf2 Signaling Pathway and Hsp70

**Figure 2:**
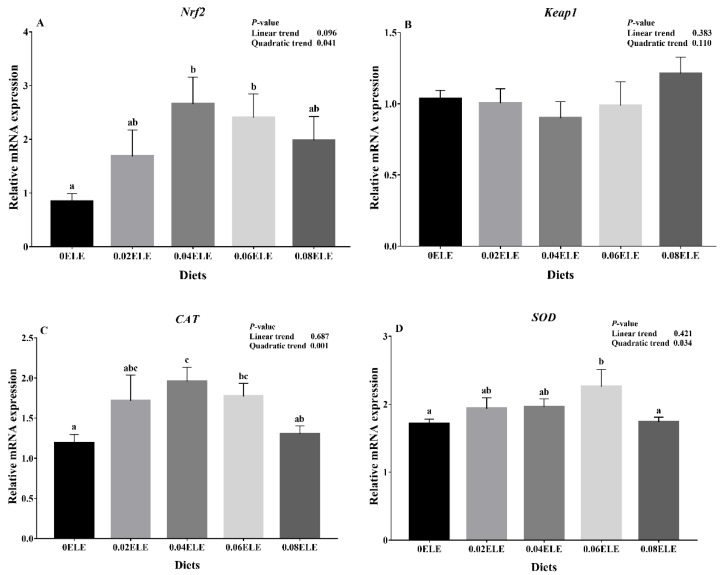

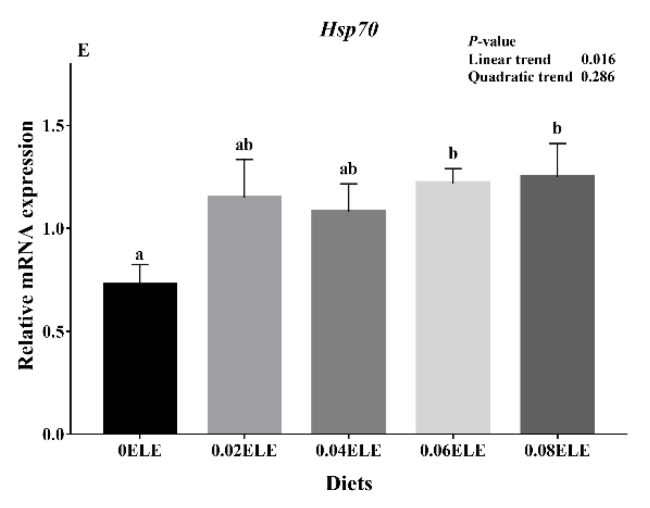
Relative expressions of *Nrf2* signaling pathway and *Hsp70* with different ELE levels. *Nrf2* (**A**); *Keap1* (**B**); *CAT* (**C**); *SOD* (**D**); *Hsp70* (**E**). Data are expressed as means ± S.E.M., value with different letters (a, b, c) are significantly different (*p* < 0.05).

Figure 2 shows the results of the relative expression of the *Nrf2* pathway and *Hsp70*. The *Nrf2*, *CAT*, and *SOD* had an open upward parabola with increasing dietary ELE inclusion levels (*p* < 0.05). Moreover, the mRNA levels of *Nrf2* in the 0.04% and 0.06% dietary ELE groups were higher than those in the control group (*p* < 0.05, Figure 2A). The *CAT* mRNA expression level in the fish fed 0.04% ELE was significantly higher than that in fish fed the control diet (*p* < 0.05, Figure 2C), and the *SOD* mRNA expression level was markedly upregulated in the fish fed 0.06% ELE (*p* < 0.05, Figure 2D). No notable changes were observed in *Keap1* mRNA levels among all treatment groups (*p* > 0.05, Figure 2B). In addition, *Hsp70* levels had a positive linear relationship (*p* < 0.05) with increasing dietary ELE inclusion levels and were higher in the 0.06% and 0.08% ELE diets than the control diet (*p* < 0.05, Figure 2E).

### 3.6. TLR2-MyD88 Signaling Pathway

**Figure 3:**
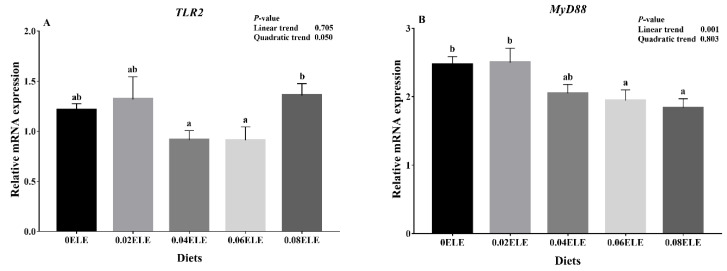
Relative expressions of *TLR2*-*MyD88* signaling pathway with different ELE levels. *TLR2* (**A**); *MyD88* (**B**). Data are expressed as means ± S.E.M., value with different letters (a, b) are significantly different (*p* < 0.05).

Figure 3A shows that no significant differences were observed in the relative expression levels of *TLR2* mRNA between the dietary ELE supplementation groups and the control group (*p* > 0.05). The *TLR2* mRNA levels in the 0.04% and 0.06% dietary ELE groups were remarkably lower than that in the 0.08% dietary ELE group (*p* < 0.05). Furthermore, the *MyD88* had a negative linear response with increasing dietary ELE inclusion levels (*p* < 0.05). Compared with the control group, 0.06% and 0.08% dietary ELE levels significantly decreased the *MyD88* mRNA expression levels (*p* < 0.05, Figure 3B).

### 3.7. Relative Expression of the Genes in the NF-κB Signaling Pathway

**Figure 4:**
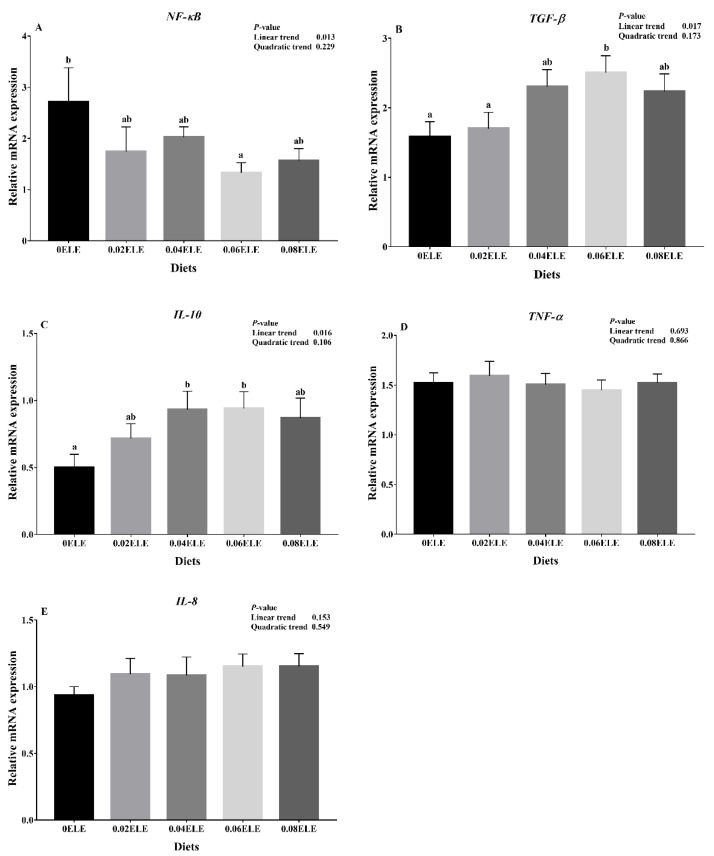
Relative expressions of *NF-κB* signaling pathway with different ELE levels. *NF-κB* (**A**); *TGF-β* (**B**); *IL-10* (**C**); *TNF-α* (**D**); *IL-8* (**E**). Data are expressed as means ± S.E.M., value with different letters (a, b) are significantly different (*p* < 0.05).

The *NF-κB* expression levels had a negative linear response with increasing dietary ELE inclusion levels (*p* < 0.05). Compared with the control group, the *NF-κB* mRNA expression level was remarkably downregulated with 0.06% dietary ELE (*p* < 0.05, Figure 4A). Conversely, the *TGF-β* expression levels had a positive linear response with increasing dietary ELE inclusion levels (*p* < 0.05), and the mRNA expression levels of *TGF-β* were higher in the 0.06% dietary ELE group than in the control group (*p* < 0.05). Similarly, the *IL-10* expression levels had a positive linear response with increasing dietary ELE inclusion levels (*p* < 0.05), the mRNA expression levels of *IL-10* increased with increasing dietary ELE from 0% to 0.06%, and the highest levels of both were found in the 0.06% dietary ELE group (*p* < 0.05, Figure 4B,C). In addition, no clear changes were found in the expression levels of the proinflammatory factors *TNF-α* and *IL-8* among all dietary treatments (*p* > 0.05, Figure 4D,E).

### 3.8. Relative Expression of the Genes in the Apoptosis Signaling Pathway

**Figure 5:**
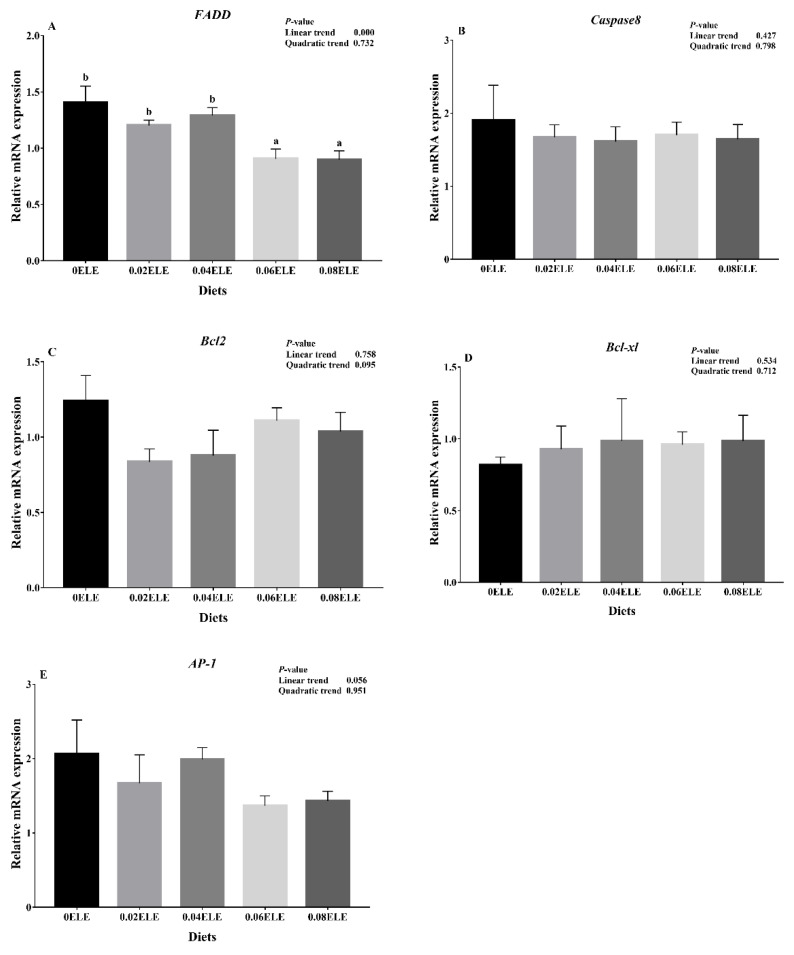
Relative expressions of apoptosis signaling pathway with different ELE levels. *FADD* (**A**); *Caspase8* (**B**); *Bcl2* (**C**); *Bcl-xl* (**D**); *AP-1* (**E**). Data are expressed as means ± S.E.M., value with different letters (a, b) are significantly different (*p* < 0.05).

The *FADD* expression levels had a negative linear response with increasing dietary ELE inclusion levels (*p* < 0.05), and at dietary ELE levels of 0.06% and 0.08%, the relative expression of *FADD* mRNA in the intestine was markedly lower than that in the control group (*p* < 0.05, Figure 5A). In addition, the expression levels of *Caspase8*, *Bcl2*, *Bcl-xl*, and *AP-1* were not influenced (*p* > 0.05, Figure 5B–E).

### 3.9. Streptococcus Agalactiae Challenge Test

**Figure 6:**
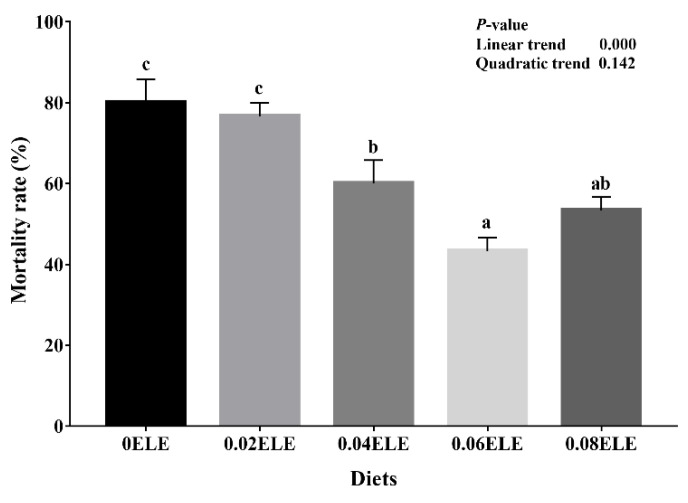
Mortality rate of GIFTs fed with different ELE levels with *Streptococcus agalactiae* challenge after 144 h. Data are expressed as means ± S.E.M., value with different letters (a, b, c) are significantly different (*p* < 0.05).

Figure 6 shows the mortality rate of the GIFT fed with different dietary ELE levels with the *Streptococcus agalactiae* challenge after 144 h. The mortality rate had a negative linear response with increasing dietary ELE inclusion levels (*p* < 0.05), and the lowest mortality rate of GIFT was observed in the fish fed 0.06% ELE (*p* < 0.05).

## 4. Discussion

### 4.1. Effects of ELE Supplementation on Growth Performance and Whole-Body Composition

In recent years, studies on aquatic animals have confirmed that ELE can promote growth performance, such as in grass carp (*Ctenopharyngodon idella*) [30], turbot (*Scophthalmus maximus* L.) [31], and large yellow croaker (*Larimichthys crocea*) [17]. However, our current results showed that dietary ELE supplementation did not improve the growth performance of GIFT. The differences in fish species and cultural environment could cause a different outcome. Since there are still relatively few studies on ELE in fish compared with mammals, more studies are needed to probe the mechanism of the effects of ELE on growth performance in aquatic animals. Furthermore, our current study showed that dietary ELE supplementation did not affect body composition, which is consistent with the findings in large yellow croaker [17].

### 4.2. Effects of ELE Supplementation on Intestinal Morphology

Intestinal morphology has a direct link to intestinal development and health status [32]. The length of the intestinal villus reflects the absorption of nutrients in the intestine, so the morphology of the intestinal villi directly reflects the growth and development of the body [33]. Our current results showed that dietary ELE supplementation did not significantly affect intestinal villus length compared with the control group, which indicated that the addition of ELE did not affect nutrient absorption in the intestine or negatively affect growth. Moreover, goblet cells maintain intestinal homeostasis by secreting mucus in the intestine to help the body absorb nutrients and defend against pathogens [34]. In this study, when the ELE level reached 0.06%, the number of goblet cells increased significantly, indicating that ELE can promote the proliferation of intestinal goblet cells to some extent. This indicates that appropriate ELE supplementation could maintain intestinal structural integrity and improve the immune barrier function of the intestine. Zhang et al. [18] proposed that ELE supplementation can improve intestinal villi structural disorders, which supports our findings.

### 4.3. Effects of ELE Supplementation on Antioxidant Status

Intestinal health is also closely related to intestinal antioxidant capacity [35,36]. The increase in the levels of relevant intestinal antioxidant enzymes and intestinal antioxidant-related genes could reflect an improvement in intestinal health [37]. In our experiment, dietary ELE supplementation significantly activated the *Nrf2* signaling pathway, which is the most important antioxidative stress defense mechanism in cells [38]. In this study, 0.04% and 0.06% dietary ELE significantly upregulated *Nrf2* mRNA expression levels. Furthermore, the downstream factors *CAT* and *SOD* were also affected by dietary ELE levels, and the highest *CAT* and *SOD* mRNA levels were present in the 0.04% and 0.06% dietary ELE groups, respectively. The results also indicated that dietary ELE supplementation could improve intestinal antioxidant capacity, which is supported by a study on channel catfish [18]. In addition, the activities of antioxidant enzymes in fish are positively correlated with the levels of their associated genes [39]. As found in this study, with the activation of antioxidant defense mechanisms, the highest CAT activity was found in the 0.04% dietary ELE group, and the highest SOD and GSH-Px activities were both present in the 0.06% dietary ELE group. This further demonstrated the efficacy of ELE to enhance antioxidant capacity. The specific reason for this result may be due to the action of the main components of ELE (chlorogenic acid [7], *E. ulmoides* flavonoids [40], and *E. ulmoides* polysaccharides [41]), which have a scavenging effect on free radicals. However, the specific mechanism needs further study. In addition, heat shock proteins (HSPs), also known as stress proteins, are preferentially synthesized after stress, among which *Hsp70* has important cellular functions, such as cytoprotective and antioxidant effects [42]. Many studies have pointed out that herbs can enhance the expression of *Hsp70* in tilapia, a mixture of Chinese herbs and a commercial probiotic *Bacillus* species could improve the expression of *Hsp70* after various stresses [43], and dietary blackberry syrup supplementation could improve the resistance of Nile tilapia to *Plesiomonas shigelloides* [44]. Likewise, in this experiment, the expression levels of *Hsp70* mRNA were elevated with dietary ELE supplementation. It was further shown that appropriate dietary ELE supplementation (0.04–0.06%) could improve the antioxidant capacity of the body. Nevertheless, the highest (0.08%) or lowest (0.02%) levels did not significantly improve the antioxidant capacity. The reason may be that the effective active ingredients of ELE have a suitable range of action, and too high or too low levels may not play their proper role.

### 4.4. Effects of ELE Supplementation on Immunocompetence

As a member of the TLR family, *TLR2* is involved in the induction of innate immune responses. [20]. *MyD88* is an important junction protein for TLRs to mediate innate immune responses, which can activate *NF-κB* in downstream signaling pathways and ultimately cause inflammatory transmitters and the release of cytokines [45]. According to a previous report on Ussuri catfish (*Pseudobagrus ussuriensis*), downregulating the mRNA expression levels of proinflammatory cytokines via the *TLR2*-*MyD88*-*NF-κB* pathway could contribute to immune competence and disease resistance [46]. In the current study, appropriate dietary ELE supplementation (0.04–0.06%) reduced the relative gene expression of *TLR2*. It is, therefore, reasonable to assume that pathogen binding to the *TLR2* protein is reduced, which in turn reduces the relative gene expression of *TLR2* [47]. As the corresponding adaptor molecules of *TLR2*, the expression levels of *MyD88* mRNA were inhibited with the addition of 0.06% ELE. In addition, the 0.06% dietary ELE group had the lowest level of *NF-κB* mRNA expression, indicating that appropriate dietary ELE supplementation might enhance GIFT immunity. Kim et al. [48] reported that *Eucommia* extract has high anti-inflammatory activity and can inhibit *NF-κB* expression. Furthermore, *NF-κB*-regulated downstream cytokines are also involved in the regulation of the immune response [49]. The present study demonstrated that 0.04–0.06% dietary ELE enhanced the mRNA expression levels of the anti-inflammatory factors *TGF-β* and *IL-10* in the GIFT intestine, while the pro-inflammatory factors *TNF-α* and *IL-8* were not affected by dietary ELE levels. The elevation of anti-inflammatory gene transcripts suggested that ELE may have a significant anti-inflammatory effect, which is consistent with a previous study on channel catfish, which showed that ELE could reduce inflammation [18]. Nevertheless, our experimental result showed that a higher level (0.08%) did not tend to improve the immune response, as reported by Huang et al. [17] where in large yellow croaker ELE exerts a suppressive effect on immune competence at high doses. Moreover, considering the economic benefits, a higher addition level (0.08%) is not recommended for GIFT. From the above experimental results, it can be inferred that appropriate dietary ELE supplementation (0.04–0.06%) could enhance GIFT immunity by suppressing the expression of relevant inflammatory factors in the *TLR2-MyD88*-*NF-κB* pathway.

In addition, plasma ALB, ALT, and AST are important nonspecific immune indicators in fish [50,51]. In our experiment, appropriate dietary ELE supplementation decreased plasma ALT and AST activities, which indicated that the hepatopancreas tissue is protected and that no significant amount of ALT and AST escapes from the cells into the blood [52]. In addition, 0.06–0.08% dietary ELE significantly increased the ALB content compared with the control diet, indicating that ELE can increase the plasma ALB content in tilapia, maintain blood osmolarity balance, promote the exchange of substances between blood and tissues, and thus improve nonspecific immunity. In addition, 0.04–0.08% dietary ELE showed increased tilapia survival rates after challenge with *Streptococcus agalactiae*. The result of the bacterial challenge test also supported our findings that ELE had positive effects on protecting tilapia from *S. agalactiae* infection.

### 4.5. Effects of ELE Supplementation on Apoptosis

The apoptotic signaling pathway is also activated by *TLR2* [22]. *TLR2* sends apoptotic signals through *MyD88* in a pathway involving *FADD* and *Caspase 8*, and the binding of *MyD88* to *FADD* is sufficient to induce apoptosis [53]. In this study, 0.06% and 0.08% dietary ELE significantly downregulated the expression levels of *FADD* mRNA, indicating that *TLR2*-mediated apoptosis was significantly inhibited by appropriate dietary ELE supplementation. In addition, *TLR2* can regulate apoptosis through the *NF-κB* pathway [54], and *NF-κB* then acts on a variety of apoptosis-related target genes, including *Bcl2*, *Bcl-xl*, and *AP-1*. In this study, these apoptosis-related genes (*Bcl2*, *Bcl-xl*, and *AP-1*) were not affected by dietary ELE levels. This could be explained by the *FADD*-mediated apoptotic pathway being the main pathway of *TLR2*-mediated apoptosis [55] rather than the *TLR2*-*NF-κB* pathway. However, the regulatory mechanisms of *TLR2*-mediated apoptotic pathways remain complex and variable and need to be further explored.

## 5. Conclusions

**Figure 7:**
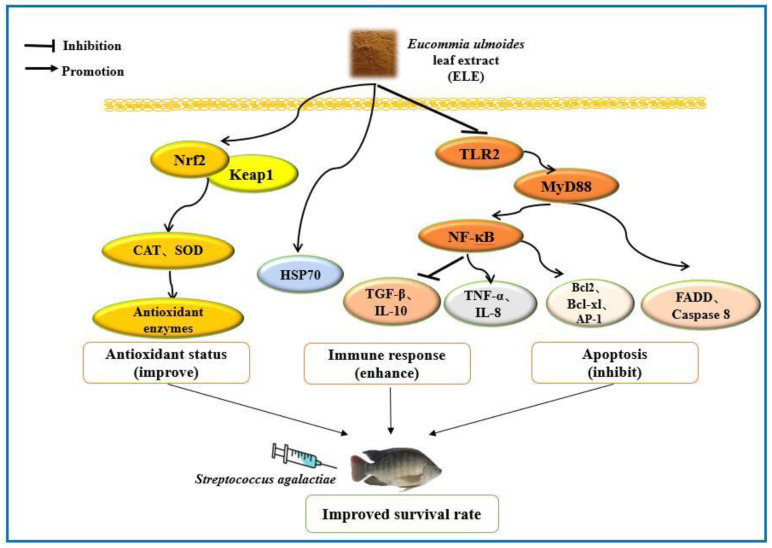
Regulation mechanism of improving health status by *Eucommia ulmoides* leaf extract (ELE) in GIFT.

In general, our current study showed that dietary ELE supplementation had no significant effect on the growth performance of GIFT. However, it was confirmed that supplementation with 0.04–0.06% ELE in the diet could promote intestinal antioxidant capacity by activating the *Nrf2* signaling pathway, enhance the immune response by suppressing the *TLR2*-*MyD88*-*NF-κB* signaling pathway, and ultimately improve the disease resistance of GIFT against *Streptococcus agalactiae* (Figure 7).

## Figures and Tables

**Table 1:** Ingredients and proximate composition of experimental diets (%, dry matter).

Ingredients	Diet 1	Diet 2	Diet 3	Diet 4	Diet 5
Fish meal ^a^	2.00	2.00	2.00	2.00	2.00
Rapeseed meal ^a^	25.00	25.00	25.00	25.00	25.00
Soybean meal ^a^	26.00	26.00	26.00	26.00	26.00
Cottonseed meal ^a^	9.00	9.00	9.00	9.00	9.00
Wheat flour ^a^	12.01	12.01	12.01	12.01	12.01
Soybean oil	2.50	2.50	2.50	2.50	2.50
Choline chloride	0.50	0.50	0.50	0.50	0.50
Vitamin C (35%)	0.05	0.05	0.05	0.05	0.05
Vitamins premix ^b^	2.00	2.00	2.00	2.00	2.00
Calcium dihydrogen phosphate	2.50	2.50	2.50	2.50	2.50
Mineral premix ^c^	2.00	2.00	2.00	2.00	2.00
Rice bran	14.05	14.05	14.05	14.05	14.05
Ethoxy quinoline	0.01	0.01	0.01	0.01	0.01
Bentonite	2.00	1.98	1.96	1.94	1.92
Methionine ^d^	0.38	0.38	0.38	0.38	0.38
ELE	0	0.02	0.04	0.06	0.08
Total	100.00	100.00	100.00	100.00	100.00
Analyzed proximate composition					
Crude protein (%)	31.55	31.74	31.73	31.88	31.78
Crude lipid (%)	7.04	7.06	7.12	7.19	7.13
Crude ash (%)	10.67	10.59	10.81	10.55	10.84

^a^ Fish meal, crude protein 65.8%, crude lipid 9.5%; Rapeseed meal, crude protein 41.3%, crude lipid 6.1%; Soybean meal, crude protein 50.8%, crude lipid 4.3%; Cottonseed meal, crude protein 53.7%, crude lipid 1.4%; Wheat flour, crude protein 13.1%, crude lipid 4.0%. They are obtained from Wuxi Tongwei feedstuffs Co., Ltd., Wuxi, China. ^b^ Vitamins premix were obtained from HANOVE Animal Health Products Co., Ltd (IU, mg/kg of premix): Vitamin A, 550000 IU; Vitamin D3, 300000 IU; Vitamin E, 3000 IU; Vitamin K3, 600 mg; Vitamin B1, 495 mg; Vitamin B2, 680 mg; Vitamin B6, 680 mg; Vitamin B12, 2.5 mg; Nicotinic acid, 2100 mg; Pantothenate, 1700 mg; Folic acid, 240 mg; Biotin, 8.5 mg; Inositol, 7000 mg; Vitamin C, 8800 mg. ^c^ Mineral premix were obtained from HANOVE Animal Health Products Co., Ltd (g/kg of premix): magnesium sulphate, 15 g; ferrous sulphate, 35 g; zinc sulphate, 13.5 g; cupric sulphate, 0.5 g; manganese sulphate, 5 g; zeolite was used as a carrier. ^d^ Methionine, obtained from Feeer Co., Ltd (Shanghai, China).

**Table 2:** The chemical analysis used in the experiment.

Items	Methods	Assay Kits/Testing Equipment
Composition of diets/whole body		
Moisture	Drying method (ID 920.36)	Electric blast drying oven (Shanghai Yiheng Scientific Instrument Co., Ltd., Shanghai, China)
Protein	Kjeldahl (ID 984.13)	Auto kieldahl apparatus: Hanon K1100 (Jinan Hanon Instruments Co., Ltd., Jinan, China)
Lipid	Soxhlet (ID 991.36)	Auto fat analyzer: Hanon SOX606 (Jinan Hanon Instruments Co., Ltd., Jinan, China)
Ash	Combustion (ID 923.03)	Muffle: XL-2A (Hangzhou Zhuochi Instrument Co., Ltd., Hangzhou, China)
Plasma parameters		
TP	International Federation of Clinical Chemistry recommended	Assay kits (TP: 105-000451-00. ALB: 105-000450-00. ALT: 105-000442-00. AST: 105-000443-00.) purchased from Mindray Medical International Ltd. (Shenzhen, China); Mindray BS-400 automatic biochemical analyzer (Mindray Medical International Ltd., Shenzhen, China).
ALB		
ALT		
AST		
Intestinal parameters related antioxidant capacity		
MDA	TBA method	Assay kits (MDA: A003-1-2. CAT: A007-1-1. SOD: A001-3-2. GSH: A006-1-1. GPx: A005-1-2.) purchased from Jian Cheng Bioengineering Institute (Nanjing, China); Spectrophotometer (Thermo Fisher Multiskan GO, Shanghai, China).
CAT	Ammonium molybdenum acid method	
SOD	WST-1 method	
GSH	Microplate method	
GPx	Colorimetric method	

**Table 3:** Real-time PCR primer sequences.

Genes ^a^	Forward primer (5′-3′)	Reverse primer (5′-3′)	Length	Accession No.
*TLR2*	GCAGCCGCTTCAAAACTCAT	GAACAAAGCCCTCAAAGCGG	105	NP_997977
*MyD88*	GTTGCGCTAAACATGAGCGT	GTCTTCTCTGTCCAGCTCCG	237	A8QMS7
*FADD*	GGCAGAAGATAACACGGCCT	ATTTGCGGCCTAGTTTTCGC	200	NP_001373289
*NF-κB*	TCACAGGGTCCTCGATGTCT	CTGGCTGTTTGGAGACAGGT	78	NP_001001839
*TGF-β*	CGTCTTCCAGCAAGCTCAGA	TCCGAAGACGCAATTCTGCT	116	NP_878293
*IL-10*	CACAACCCCAATCGACTCCA	GAGCAAATCAAGCTCCCCCA	175	NP_001018621
*IL-8*	GGAAGACCTGCCTCAATCCC	GGGGCGGAGGTAGAATTTGG	118	XP_001342606
*TNF-α*	GCAATCCGCTCAATCTGCAC	GCAGCGCCGAGGTAAATAGT	74	NP_998024
*Caspase8*	ACCAGGACCTGCTGTCATTG	TATCTGGAGATGCGCTGCTG	160	XP_685430
*Bcl-2*	GCGCTTCAACGCAGTCATAG	GCAGCTAGACCAAAGACCGT	291	XP_001341214
*Bcl-xl*	CAAGGAGGATGGGAACGCTT	TTCTGTGCAATGAGTCCCCC	146	NP_571882
*AP-1*	CGTGAGTGTCACCTCGACTC	GTCCTCATAAACCGGCGACT	127	NP_956281
*Nrf2*	CTGCCGTAAACGCAAGATGG	ATCCGTTGACTGCTGAAGGG	287	NM_182889.1
*Keap1*	GGAAGTCACCCTTCGAGACG	AGAGGACGTGAAGAACGCAG	107	NM_182864.2
*CAT*	GGAAGAGGATGACGAAGAG	GTTACGGCGAGATGATGT	232	NP_570987
*SOD*	ACAGAAGAGAAGTATCAGGAG	CACCGTAACAGCAGACAT	228	NP_956270
*Hsp70*	TCCATCACAAGGGCACGTTT	CAGGGCTTTCTCAACTGGGT	78	Q91233.1
*β-actin*	ACCCCATTGAGCACGGTATT	GCTCCTCAGGGGCAACTCTC	96	KJ126772.1

^a^*TLR2*, Toll like receptor 2; *MyD88*, myeloid differentiation factor 8; *FADD*, Fas-associating protein with a novel death domain; *NF-κB*, Nuclear factor Kappa B; *TGF-β*, Transforming growth factor-β; *IL-10*, Interleukin 10; *IL-8*, Interleukin 8; *TNF-α*, Tumor necrosis factor-α; *Caspase8*, Cysteine-requiring aspartate protease 8; *Bcl-2*, B-cell lymphoma-2; *Bcl-xl*, B-cell lymphoma-xl; *AP-1*, Activating protein-1; *Nrf2*, Nuclear factor erythroid 2-related factor 2; *Keap1*, Kelch-like ECH-associated protein1; *CAT*, Catalase; *SOD*, Superoxide dismutase; *Hsp70*, Heat shock protein 70.

**Table 4:** Growth performance of the GIFT fed with different diets.

*Eucommia ulmoides* Leaf Extract (%)	IBW (g) ^a^	FBW (g) ^b^	FCR ^c^	WGR (%) ^d^	SGR (% Day^−1^) ^e^	SR (%) ^f^
0	12.08 ± 0.04	73.75 ± 4.39	0.59 ± 0.04	510.4 ± 37.00	3.47 ± 0.12	93.3 ± 6.67
0.02	12.08 ± 0.04	72.32 ± 2.43	0.59 ± 0.02	498.5 ± 20.43	3.44 ± 0.07	100.0 ± 0.00
0.04	12.02 ± 0.03	74.22 ± 3.33	0.59 ± 0.03	517.7 ± 28.94	3.50 ± 0.09	100.0 ± 0.00
0.06	12.03 ± 0.02	71.93 ± 2.19	0.62 ± 0.02	497.7 ± 17.53	3.44 ± 0.06	100.0 ± 0.00
0.08	12.03 ± 0.04	69.38 ± 0.43	0.63 ± 0.00	506.7 ± 26.46	3.46 ± 0.08	100.0 ± 0.00
*p*-value						
Linear trend	0.241	0.339	0.278	0.393	0.429	0.188
Quadratic trend	0.572	0.564	0.661	0.540	0.516	0.260

Data are expressed as means with SEM. Values with different superscripts are significantly different (*p* < 0.05). ^a^ IBW: initial body weight. ^b^ FBW: final body weight. ^c^ Feed conversion ratio (FCR) = dry feed fed (g)/(final body weight (g)—initial body weight (g)). ^d^ Weight gain rate (WGR) (%) = 100 × (final body weight (g—initial body weight (g))/initial body weight (g). ^e^ Specific growth rate (SGR) (% day^−1^) = 100 × [(In (final body weight (g))—In (initial body weight (g)))/days]. ^f^ Survival rate (SR) (%) = 100 × (survival fish number/total fish number).

**Table 5:** Whole-body composition of the GIFT fed with different diets.

Eucommia ulmoides Leaf Extract (%)	Moisture (%)	Protein (%)	Lipid (%)	Ash (%)
0	74.53 ± 0.57	14.47 ± 0.76	5.20 ± 0.23	4.04 ± 0.13
0.02	75.14 ± 0.37	14.36 ± 0.20	4.45 ± 0.25	3.82 ± 0.11
0.04	74.00 ± 0.84	14.69 ± 0.32	5.34 ± 0.93	4.10 ± 0.29
0.06	73.98 ± 0.59	15.02 ± 0.43	4.98 ± 0.45	3.86 ± 0.06
0.08	73.55 ± 0.31	14.99 ± 0.19	5.95 ± 0.27	3.73 ± 0.05
p-value				
Linear trend	0.112	0.289	0.200	0.086
Quadratic trend	0.656	0.937	0.297	0.330

Data are expressed as means with SEM. Values with different superscripts are significantly different (*p* < 0.05).

**Table 6:** Plasma parameters of the GIFT fed with different diets.

*Eucommia ulmoides* Leaf Extract (%)	TP (g/L)	ALB (g/L)	ALT (U/L)	AST (U/L)
0	31.25 ± 0.99	14.87 ± 0.53 ^a^	36.91 ± 2.62 ^b^	90.12 ± 8.74 ^b^
0.02	30.05 ± 1.22	14.71 ± 0.29 ^a^	35.16 ± 3.34 ^ab^	81.67 ± 8.00 ^b^
0.04	29.56 ± 1.15	14.96 ± 0.59 ^ab^	30.52 ± 4.92 ^ab^	73.60 ± 8.96 ^ab^
0.06	33.84 ± 1.96	16.61 ± 0.71 ^b^	31.73 ± 3.40 ^ab^	64.60 ± 5.27 ^a^
0.08	33.77 ± 1.50	16.19 ± 0.50 ^ab^	25.14 ± 2.26 ^a^	62.57 ± 7.68 ^a^
*p*-value				
Linear trend	0.054	0.009	0.000	0.003
Quadratic trend	0.188	0.528	0.644	0.656

Data are expressed as means with SEM. Means with the same letters or absence of letters indicate not significantly different between treatments (*p* > 0.05). Values with different superscripts (a, b) are significantly different (*p* < 0.05).

**Table 7:** Intestinal antioxidant enzyme activities of the GIFT fed with different diets.

*Eucommia ulmoides* leaf extract (%)	CAT (U/mgprot)	SOD (U/mgprot)	MDA (nmol/mL)	GSH (umol/gprot)	GSH-Px (U/mgprot)
0	1.60 ± 0.11 ^a^	0.34 ± 0.04 ^a^	0.97 ± 0.09	56.88 ± 2.59	2.37 ± 0.32 ^a^
0.02	1.68 ± 0.09 ^ab^	0.37 ± 0.04 ^ab^	0.84 ± 0.07	59.90 ± 3.16	2.82 ± 0.41 ^ab^
0.04	2.03 ± 0.08 ^c^	0.41 ± 0.04 ^ab^	0.91 ± 0.06	69.59 ± 5.71	3.71 ± 0.39 ^bc^
0.06	1.94 ± 0.10 ^bc^	0.48 ± 0.03 ^b^	0.88 ± 0.08	67.59 ± 4.17	4.26 ± 0.58 ^c^
0.08	1.77 ± 0.12 ^abc^	0.38 ± 0.04 ^ab^	0.92 ± 0.10	58.95 ± 5.87	3.68 ± 0.42 ^bc^
*p*-value					
Linear trend	0.066	0.142	0.886	0.411	0.005
Quadratic trend	0.018	0.047	0.441	0.044	0.147

Data are expressed as means with SEM. Means with the same letters or absence of letters indicate not significantly different between treatments (*p* > 0.05). Values with different superscripts (a, b, c) are significantly different (*p* < 0.05).

**Table 8:** The effects of ELE on the intestinal morphology of the GIFT.

Parameters	*Eucommia ulmoides* Leaf Extract (%)				
	0	0.02	0.04	0.06	0.08
Number of goblet cells	9.6 ± 0.7 ^a^	9.4 ± 1.3 ^a^	13.6 ± 1.4 ^ab^	15.4 ± 2.2 ^b^	12.2 ± 2.4 ^ab^
Villus length (mm)	0.72 ± 0.07 ^ab^	0.60 ± 0.01 ^a^	0.80 ± 0.07 ^b^	0.81 ± 0.04 ^b^	0.78 ± 0.07 ^b^

Data are expressed as means with SEM. Means with the same letters or absence of letters indicate not significantly different between treatments (*p* > 0.05). Values with different superscripts (a, b) are significantly different (*p* < 0.05).

## Data Availability

All of the data is contained within the article.
